# Bayesian multilevel analysis of determinants of acute respiratory infection in children under the age of five years in Ethiopia

**DOI:** 10.1186/s12887-022-03187-4

**Published:** 2022-03-10

**Authors:** Amanuel Merera, Tilahun Asena, Mebratu Senbeta

**Affiliations:** 1 Department of Statistics, Mizan-Tepi University, Mizan-Tepi, Ethiopia; 2 Department of Statistics, Arba Minch University, Arba Minch, Ethiopia; 3 Department of Economics, Arba Minch University, Arba Minch, Ethiopia

**Keywords:** Acute respiratory infections, Bayesian multilevel, MCMC

## Abstract

**Background:**

Acute respiratory tract infection (ARI) is one of the leading causes of illness and mortality in children under the age of five worldwide. Pneumonia, which is caused by a respiratory tract infection, kills about 1.9 million children under the age of 5 years around the world. The majority of these deaths occur in underdeveloped countries. According to the 2016 Ethiopia Demographic and Health Survey (EDHS), the prevalence rate of ARI in Ethiopia was 7%. Prevalence is defined as the number of infectious diseases present at a given period in relation to the total number of children under the age of five who have been exposed to ARI. The goal of this study was to determine the risk factors for acute respiratory infection among children under the age of five in Ethiopia.

**Methods:**

To provide representative samples of the population, a community-based cross-sectional sampling scheme was designed. Bayesian multilevel approach was employed to assess factors associated with the prevalence of ARI among children under age five in Ethiopia. The data was collected from 10,641 children under the age of 5 years out of which 9918 children were considered in this study.

**Results:**

The ARI prevalence rate in children under the age of 5 years was assessed to be 8.4%, somewhat higher than the country’s anticipated prevalence rate. Children whose mothers did not have a high level of education had the highest prevalence of ARI. The key health, environmental, and nutritional factors influencing the proportion of children with ARI differed by area. Tigray (15.3%) and Oromia (14.4%) had the highest prevalence of ARI, while Benishangul Gumuz had the lowest prevalence (2.6%). The use of vitamin A was investigated, and the results revealed that roughly 43.1% of those who received vitamin A had the lowest prevalence of ARI (7.7%) as compared to those who did not receive vitamin A. Diarrhea affected 11.1% of children under the age of five, with the highest frequency of ARI (24.6%) and the highest prevalence of ARI reported in children whose drinking water source was unprotected/unimproved (9.4%).

**Conclusions:**

The prevalence of ARI among children under the age of 5 years was found to be strongly affected by the child’s age, household wealth index, mother’s educational level, vitamin A supplement, history of diarrhea, maternal work, stunting, and drinking water source. The study also found that the incidence of ARI varies significantly between and within Ethiopian areas. When intending to improve the health status of Ethiopian children, those predictive variables should be taken into consideration.

**Supplementary Information:**

The online version contains supplementary material available at 10.1186/s12887-022-03187-4.

## Background

Acute respiratory tract infection is one of the leading causes of illness and mortality among children under the age of five. Pneumonia caused by a respiratory tract infection is the leading cause of death in children under the age of five worldwide, accounting for nearly 15% of all deaths. Approximately half of these deaths occur in Sub-Saharan Africa [1]. Lower respiratory infections are the fifth leading cause of death worldwide, as well as the top cause of death among children under the age of five [2].

Low and middle-income countries bear the brunt of the high incidence and prevalence of ARIs in comparison to high-income ones. According to the WHO, there were 2.1 million ARI-related deaths in children under the age of 5 years old per year (excluding deaths caused by measles, pertussis, and newborn mortality). Every year, 10.8 million children die as a result of ARI [3]. According to estimates, nearly 1.9 million children died from ARI in the year 2000, with 70% of those deaths occurring in Africa and Southeast Asia [4].

Pneumonia is an illness that inflames the air sacs in one or both lungs and causes roughly 1.9 million (1.6 - 2.2 million) fatalities in children under the age of 5 years each year, with 90% of these deaths occurring in developing countries [5]. In low- and middle-income countries, acute respiratory infections are the leading cause of illness and mortality among children under the age of five (ARIs). Acute lower respiratory infections (ALRI) such as pneumonia and bronchitis affect roughly 126–156 million children worldwide each year, resulting in about 1.4 million deaths.

Africa and Southeast Asia account for more than 95% of these deaths [6–8]. According to several studies, the leading causes of mortality in poor countries were a lack of health care services, poverty, and a lack of education. Children in underdeveloped nations are ten to fifty times more likely than children in industrialized countries to die from ARI [9]. Despite the fact that Ethiopia met the fourth Millennium Development Goal 3 years ago by reducing under-five mortality by 67% from the 1990 estimate, around 190,000 children still die each year from various reasons.

According to WHO estimates, ARI caused 18% of fatalities among children under the age of five in 2014 [10]. According to the Integrated Community Case Management Survey in Amhara, SNNP, and Tigray Regions [11], around 19% of Ethiopian children had ARI in the previous 2 weeks prior to the survey.

Prevalence is commonly used to express the populations health status; however, prevalence is defined as the proportion of persons in a population who have a particular disease or attribute at a specified point in time[12]. Therefore, the prevalence of ARI was reported to be 7% in the 2016 EDHS [13]. For demographic and health survey investigations, several studies have been undertaken, including possible comparisons between Bayesian and classical/frequents techniques [14–16]. The behavior of a parameter is handled differently in both ways. A parameter is treated as a fixed value in the frequents method, whereas it is treated as a random variable with a distribution in the Bayesian ideology. In this study, the purpose of using a multilevel model in a Bayesian framework is to evaluate regional differences. To examine the variations, frequentist multilevel models are employed, although prior distributions to the likelihood are not included. As a result, the classical model is naturally less accurate than Bayesian method models, because all information in the classical model was gained solely from likelihood, whereas in the Bayesian approach, prior information and likelihood were combined. Furthermore, Bayesian statistical analysis has high power and computes the posterior distribution, which excludes prior information from the present data and inference is based on the posterior distribution [17–19]. This prior improves the accuracy and reliability of the estimations. Although the application of Bayesian statistics sounds appealing to researchers, it has remained theoretically defined for over a century due to difficulties in integrating the denominator in Bayes theorem. Thanks to simulation based MCMC methods, the approach has been valued to have numerical meaning with the efficient estimation of the application in any field, with some limitations such as the burden of time in approximating the posterior and the convergence problem [20, 21].

Researchers from all around the world used several traditional methodologies to identify major risk factors for acute respiratory infections (ARIs) in children under the age of five [22, 23], but they were unable to determine whether or not there were geographical differences. The traditional approach fails to account for unmeasured random heterogeneity effects in ARI among children under the age of five, as well as the geographic inequalities in the prevalence of children under the age of five among areas. These statistical methods fail to account for group variability and do not take into account the interdependence of observations within the same group/cluster. Although the conventional strategy uses an iterative approach to fit regression models, convergence may be difficult to accomplish in some circumstances due to this iterative approach. Bayesian approaches are gaining favor in data analysis due to the robustness and accuracy of the results they give. The goal of this research is to look into the factors that influence the prevalence of ARI morbidity in children under the age of five, as well as to look into the differences in ARI within and between Ethiopian regions.

## Methods

### Study design and setting

**Fig. 1:**
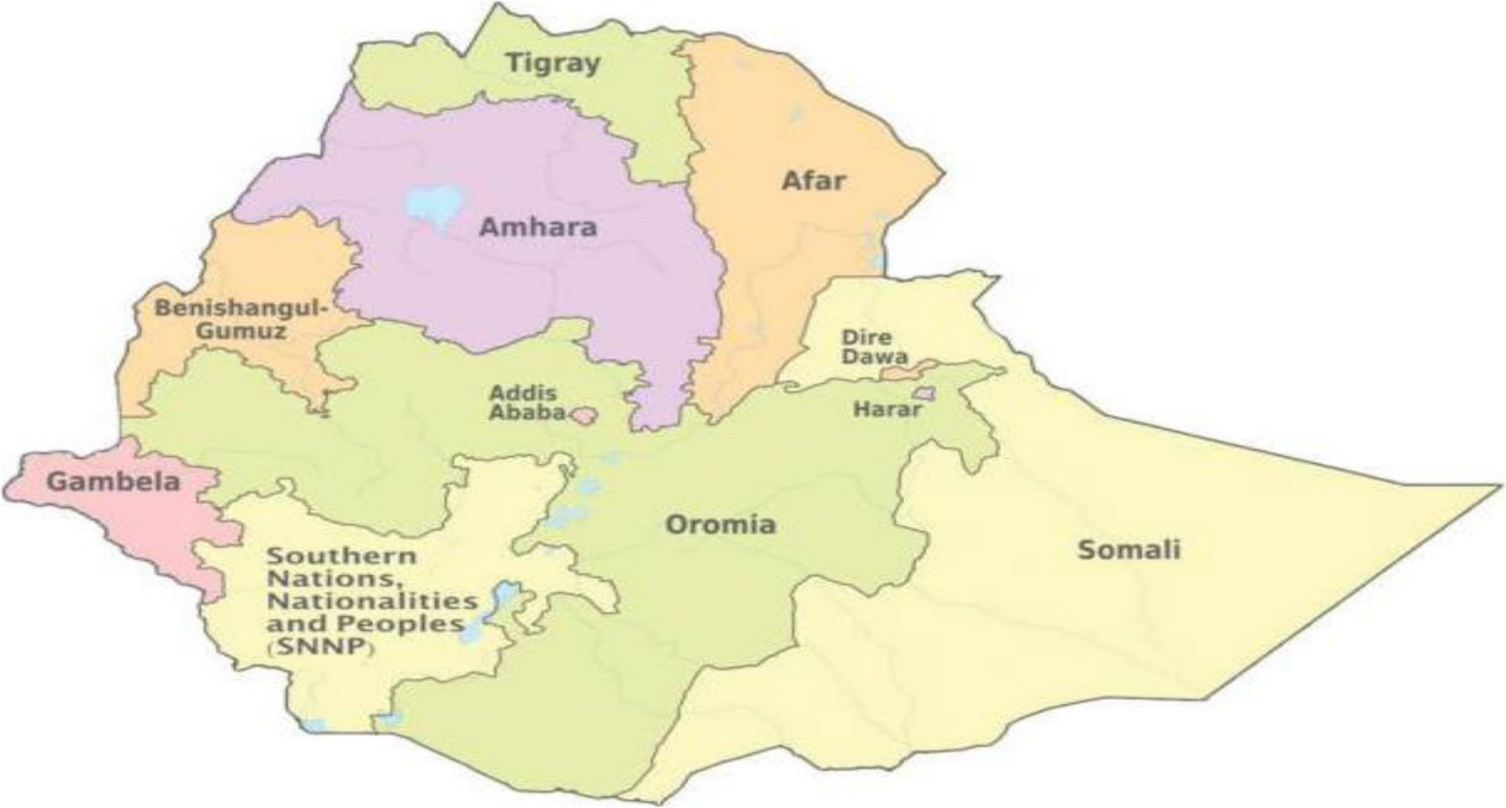
Regional state maps of Ethiopia. (Map source: Maps of Ethiopia’s regional states.) Image courtesy of https://www.ethiovisit.com/addis-ababa/65/ (accessed September 20,2021))

From January 18 to June 27, 2016, a cross-sectional investigation was undertaken. The data for this study came from the Ethiopian Demographic and Health Survey, which was completed in 2016. The 2016 EDHS was Ethiopia’s fourth survey as part of the global Demographic and Health Surveys programme. It was carried out at the request of the Federal Ministry of Health by the Central Statistical Agency (CSA) (FMoH). A standardized and pre-tested questionnaire was utilized to collect data. Interviewers used tablet computers to record responses during the 2016 EDHS interviews. The tablets were outfitted with Bluetooth technology to enable remote electronic file transfer (transferring assignment sheets from team supervisors to interviewees and completed copies from interviewers to supervisors) [13]. In 2016, the EDHS selected a representative sample of approximately 18,008 households from 624 clusters across 9 regions and 2 administrative cities (See Fig. 1).

The 2016 EDHS sample was selected using a two-stage cluster design, with census enumeration areas (EAs) serving as sampling units in the first stage. A typical two-level stratification involves first stratifying the population by region and then by urban-rural within each region. There were 645 EAs in the sample (202 in urban areas and 443 in rural areas). The second stage of sampling in the sampling procedure was households. Equal probability systematic sampling using proportional to EAs was used to conduct a complete listing of households in each of the 645 selected enumeration areas.

**Fig. 2:**
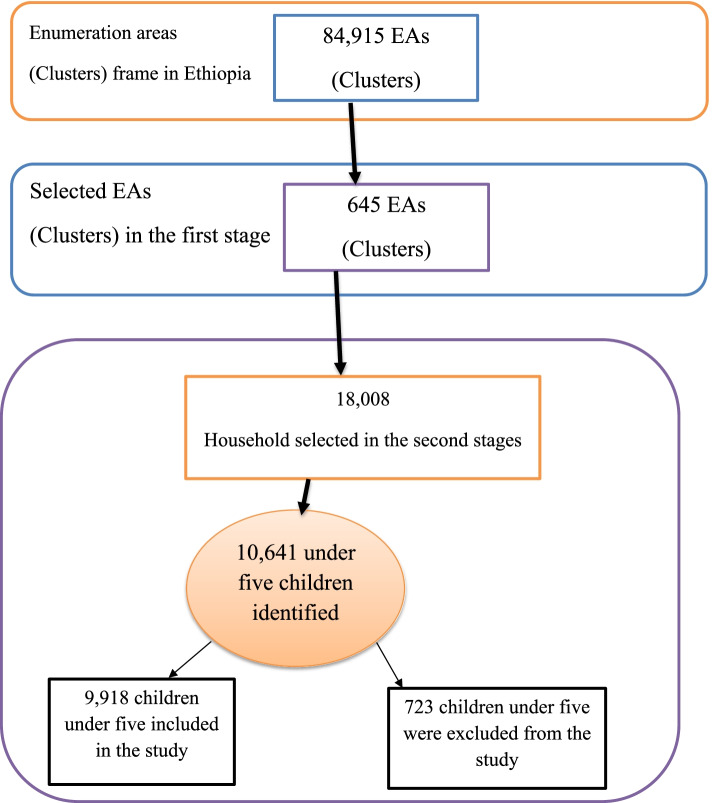
Depicts the sampling procedure for the study predictors of ARI among Ethiopian children under the age of five (*n* = 9918)

All women between the ages of 15-49 who had at least one child in the 5 years preceding the survey were eligible to take part. This study’s sample size would be 10,641 children aged five and under [13]. Because the EDHS data contains many missing values across some variables, a total of 9918 under-five children were included in this study after those missing values were removed (See Fig. 2: The conceptual frame work of sampling procedure).

### Criteria for inclusion and exclusion

Children under the age of five who resided with their families and whose parents agreed to participate were included in the study, but children whose mothers supplied inadequate information or children who were very ill during data collection were excluded.

### Measurement of variables

The presence or absence of ARI in a child under the age of five was the outcome variable, which was coded with a value of zero to indicate absence of ARI and one to indicate presence of ARI. In the last 2 weeks, a cough and short, quick breaths were required for a child to be diagnosed as having ARI, which were quantified based on mothers’ complaints regarding the symptoms of these diseases [13]. We used selected variables for investigation based on the original coding as it appears in the EDHS, depending on the availability of the datasets and their theoretical link known from the literature [13, 23–26]. Many variables, on the other hand, were derived by adding two or more variables together or by recording the values of individual variables. As potential predictors of ARI, the following variables were chosen: Age of child (less than 6 months, 6–11, 12–23, 24–35, 36–47, 48–59); maternal age (15–19, 20–34, 35–49); place of residence (urban, rural); region (Tigray, Afar, Amhara, Oromia, Somalia, Benishangul Gumuz, SNNPR, Gambela, Harari); maternal education level (no education, primary, secondary and above); Cooking fuel (dirty, clean); wealth quintile (poorest, poorer middle, richer, richest); maternal work (not working, working); and child sex (male, female). Breast-feeding (Never breast-feed, ever breast-feed not currently, still breast-feeding); Vitamin A supplementation (yes, no), recent diarrhea (no, yes), number of living children (1-3 child,4-6 child, above 6). Drinking water sources were recoded as “improved” or “unimproved” [27]. Children’s nutritional status was assessed by calculating z-scores for “height-for-age (stunting)” and “weight-for-height (wasting)” using WHO-recommended child physical growth indicators [13, 28]. Children were classified as stunted or wasted if their z-score for each nutritional status was two standard deviations lower than the WHO reference population median [13, 28].

### Statistical analysis

The statistical analysis for this study was based on a Bayesian multilevel logistic model. This model was chosen because the data collection procedure is a hierarchical level or structures, which means the levels are nested one another. The following parameters were estimated using Markov Chain Monte Carlo (MCMC) methods: The Markov chain Monte-Carlo (MCMC) method is a general simulation technique used to sample posterior distributions and calculate posterior quantities of interest [28]. Because the sampling procedure of MCMC approaches is quite intensive, but it is bias-free, these methods are preferred when precise results are desired, regardless of the time required [29].

It is critical to know whether a series of MCMC iterations has converged or reached its stationary distribution. Examining MCMC convergence will necessitate considering a different strategy for locating poorly sampled Markov chains. Time Series Plots, Kernel Density Plots, Monte Carlo Standard Error (MCSE), Effective Sample Size (ESS), and Partial Autocorrelation Function (PACF) were used in our study for variable convergence testing. In this study, three models were fitted: Model 1 has no covariates; Model 2 has fixed effects and regional random effects; and Model 3 incorporates factors from Model 2 as well as variables in Model 2 that have a significant impact on ARI in children under the age of five by monitoring their respective area effect. The Deviance Information Criterion is a common statistic used for comparing models in a Bayesian framework. Finally, we calculated the posterior odds ratio (OR) and 95% Bayesian credible intervals (CI) for each variable in the overall model using DIC (Model 3). Statistical significance was calculated based on the absence of 1.0 in the 95% confidence interval. (See Supplementary File 1 for more information).

## Results

This study was conducted to investigate the determinant factors associated with acute respiratory infections in children under the age of five, and the overall ARI prevalence rate was 8.4%, which was slightly higher than the country’s estimated prevalence level. Males had the highest prevalence of ARI (8.5%). Approximately (81.1%) of children under the age of five were born to mothers who lived in a rural area and had a higher prevalence of ARI (8.9%). A low-income household had the highest incidence of ARI among children under the age of five (10.3%). Furthermore, the highest prevalence of ARI was found in children whose mothers had no education (9.6%). (56.9%) of the 9918 children under the age of five (Fig. 2) had not recently received vitamin A, with the highest proportion having ARI (9%). Furthermore, 11.1% of children under the age of five recently experienced diarrheas, with the highest prevalence of ARI (24.6%). The child with the highest rate of ARI had a drinking water source that had not been protected or improved (9.4%). As shown in Table 1, Tigray and Oromia had the highest rates of ARI (15.3 and 14.4%, respectively), while Benishangul Gumuz had the lowest (2.6%). It implies that there are regional variations.

**Table 1:** Distribution of Demographic, Socio-economic, Health, Environmental and Nutritional related Factors on prevalence of ARI among children under five in Ethiopia from January 18 to June 27, 2016 (*n* = 9918)

		ARI STATUS		
Variables	Categories	Had no ARI	Had ARI	*P*-value
		Count (%)	Count (%)	
Sex of child	Male	4632(91.5)	430(8.5)	0.839
	Female	4449(91.6)	407(8.4)	
Child age	< 6 months	1022(91.6)	94(8.4)	
	6-11 months	891(87.7)	125(12.3)	< 0.001*
	12-23 months	1704(88.6)	219(11.4)	
	24-35 months	1748(91.7)	158(8.3)	
	36-47 months	1755(92.6)	141(7.4)	
	48-59 months	1961(95.1)	100(4.9)	
Mothers age	15-19	336(91.3)	32(8.7)	
	20-34	6565(91.5)	614(8.5)	0.740
	35-49	2180(91.9)	191(8.1)	
Wealth index	Poorest	3325(90.5)	350(9.5)	< 0.001*
	Poorer	1493(89.7)	171(10.3)	
	Middle	1272(92.2)	108(7.8)	
	Richer	1134(93.3)	81(6.7)	
	Richest	1857(93.6)	127(6.4)	
Place of residence	Rural	7325(91.1)	720(8.9)	< 0.001*
	Urban	1756(93.7)	117(6.3)	
Mother education	No education	5806(91.4)	548(9.6)	< 0.001*
	Primary	2261(90.4)	240(8.6)	
	Secondary and above	1012(95.2)	49(4.8)	
Mother occupation	Not working	6583(91.9)	580(8.1)	0.048*
	Working	2498(90.7)	257(9.3)	
Vitamin A supplement	No	5134(91)	505(9)	0.034*
	Yes	3947(92.3)	332(7.7)	
Breast feeding	Never breast feed	358(94.5)	21(5.5)	< 0.001*
	ever breast feed not currently	4932(93.3)	357(6.7)	
	Still breast feed	3791(89.2)	459(10.8)	
History of diarrhea	No	8249(93.6)	565(6.4)	< 0.001*
	Yes	832(75.4)	272(24.6)	
Type of cooking fuel	Unclean/unsafe	8599(91.4)	810(8.6)	0.009*
	Clean/Safe	482(94.7)	27(5.3)	
Wasting	Not wasted	8058(91.8)	723(8.2)	0.041*
	Wasted	1023(90)	114(10)	
Stunting	Not stunted	5880(92.8)	458(7.2)	< 0.001*
	Stunted	3201(89.4)	379(10.6)	
Number of	1-3 child	4551(92)	397(8)	0.309
child	4-6 child	3226(91.1)	317(8.9)	
	above 6 child	1304(91.4)	123(8.6)	
Source of water	Unprotected	5182(90.6)	537(9.4)	0.002*
	Protected	3899(92.8)	300(7.2)	
Region	Tigray	835(84.7)	151(15.3)	< 0.001*
	Affar	892(92.4)	73(7.6)	
	Amhara	824(89.2)	100(10.8)	
	Oromia	1272(85.6)	214(14.4)	
	Somale	1329(95.7)	60(4.3)	
	Benishangul gumuz	793(97.4)	21(2.6)	
	SNNPS	1081(90.3)	116(9.7)	
	Gambela	624(95.4)	30(4.6)	
	Harari	543(97.3)	15(2.7)	
	Addis Ababa	411(94.1)	26(5.9)	
	Dire Dawa	477(93.9)	31(6.1)	

*significant, Pearson chi-square test was used to calculate the *p*-values

### Bayesian multilevel logistic regression analysis

The Bayesian method provides parameter estimates by sampling them from their posterior distributions using an MCMC method. For the fixed effects *α*, the Metropolis-Hasting algorithm was implemented using a non-informative uniform prior distribution with a scale parameter and an inverse gamma distribution with *α* and *θ* for the random effect [30].

### Bayesian multilevel logistic regression model comparison

The comparison of the fit of Bayesian multilevel logistic regression models using the summary of the fitted model is shown in Supplementary file 2 of Tables A1 and A2. The model with the smallest DIC is the best model for the data set, so the result shows that model 3 was a better fit in any combination of variables in the data set than the other models.

Model 1: The empty model has no explanatory variables and can be thought of as a parametric version of assessing heterogeneity among regions in terms of ARI status of children under the age of five. The variance of the random factor is significant, indicating that ARI varies by region. Model 2: Model 2: The random intercept model allows the intercept to vary across regions after controlling for ARI covariates. The random intercept model results showed that the random intercept *β*_0*j*_ is significant, implying that the average prevalence of ARI varies by region. The results show that the variance of the random effect is significant, indicating that ARI varies by region. Model 3 (Random Coefficient Bayesian Multilevel) fit the data the best, according to the model comparisons. (For more information, see Supplementary file 2).

### Model diagnostic check

Following the development of the model, the model’s effectiveness is assessed using goodness of fit and convergence monitoring. The diagnostic is carried out by calculating quintiles (the 2.5 and 97.5% quintiles) that will form a central interval estimate. Furthermore, the trace of the chains, autocorrelations (AC), and partial autocorrelations (PAC) functions at iteration t and t-k after accounting for iterations − 1,…..., t-(k-1), where k is parameters in iterations and Monte Carlo standard errors (MCSE) are investigated for each of the posterior distributions of the parameter in the model diagnostics.

The convergence of the posterior estimate was tested using effective sample size estimates greater than 200 [31]. We save more samples to obtain an efficient posterior estimate, and our posterior estimates become more accurate. This has been presented in various plots for various variables. Supplementary file 2 includes, for example, convergence plots for the wealth index and diarrhea.

We expected our model to be good enough to fit our data because the traces plots, AC and PAC functions are less correlated and the MCSE is close to zero. The MLwiN manual [32] contains a detailed method for parameter estimation and model diagnostics using MCMC simulation methods. The MC error for each significant variable is less than 5% times its standard deviation for all of our variables. The convergence and accuracy of posterior estimates have been achieved, and the model is suitable for estimating the posterior statistics.

### The random coefficient Bayesian multilevel Model’s results

According to the results of the Multilevel Random Coefficient Model, age of child, wealth index of household, maternal education level, vitamin A supplement, had diarrhea recently, maternal working status, stunting, and source of drinking water were found to be significant, indicating strong effects on ARIs of under five children and also contributing to variations across regional states in Ethiopia. The odds of having ARI were 40.2% lower in children under the age of five in the 48–59-month age group compared to children under the age of 6 months. Children under the age of five who had mothers who had completed secondary and higher education levels were 33.5% less likely to develop ARI than children under the age of five whose mothers had no education.

According to this study, vitamin A consumption by children in the previous 6 months had a significant effect on ARIs. A child who had recently received vitamin A was 16.6% less likely to suffer from ARI than a child who had not recently received vitamin A. Indeed, children under the age of five who had diarrhea recently were 4.284 times more likely to have ARI than children who had not had diarrhea recently.

Maternal occupation also has a statistically significant association with ARI; thus, the odds of children whose mothers worked increased by 30.9% when compared to mothers who did not work. Another finding from Table 2 revealed that the chronic nutritional status of children explained by stunting was included in the model and has a significant effect on ARI. Under-five children who were stunted were 59.5% more likely to experience ARI than children who were not stunted.

**Table 2:** Bayesian Multilevel Logistic Regression of Random Coefficients

Variables	Category	Posterior mean	MC error	SD	OR	OR(95% CI)
–	Intercept	−3.305	0.0057	0.353	0.0376	0.018-0.077
Sex	Male (ref)					
	Female	0.011	0.0003	0.081	1.016	0.867-1.185
Child age in months	< 6 (ref)					
	6-11	0.259	0.0008	0.163	1.295	0.944-1.784
	12-23	0.095	0.0008	0.151	1.099	0.823-1.474
	24-35	−0.155	0.001	0.175	0.856	0.613-1.206
	36-47	−0.164	0.0011	0.190	0.849	0.582-1.212
	48-59	−0.514	0.0011	0.201	0.598	0.403-0.878
Maternal age	15-19(ref)					
	20-34	0.142	0.0023	0.221	1.153	0.754-1.791
	35-49	0.029	0.002	0.251	1.029	0.630-1.702
Wealth index of household	Poorest (ref)					
	Poorer	−0.222	0.0005	0.125	0.800	0.624-1.021
	Middle	−0.632	0.0006	0.147	0.532	0.396-0.709
	Richer	−0.877	0.0015	0.245	0.416	0.242-0.647
	Richest	−0.638	0.001	0.210	0.528	0.349-0.798
Place of residence	Rural (ref)					
	Urban	0.264	0.0011	0.225	1.302	0.840-2.032
Breast feeding	Never breast feed (ref)					
	Ever breast feed, not currently	0.188	0.0024	0.250	1.207	0.761-2.007
	Still breast feed	0.337	0.0028	0.258	1.400	0.854-2.387
Maternal education	No education (ref)					
	Primary	0.140	0.0004	0.106	1.150	0.938-1.419
	Secondary and higher	−0.454	0.0007	0.205	0.635	0.417-0.935
Vitamin A supplement	No (ref)					
	Yes	−0.182	0.0003	0.088	0.834	0.698-0.989
Had diarrhea recently	No (ref)					
	Yes	1.455	0.0003	0.099	4.284	3.525-5.202
Fuel type	Unclean/unsafe (ref)					
	Safe/clean	−0.062	0.001	0.269	0.939	0.545-1.560
Maternal work	Not working (ref)					
	Working	0.270	0.0007	0.137	1.309	1.011-1.716
Wasting	No (ref)					
	Yes	0.125	0.0004	0.122	1.133	0.889-1.433
Stunting	No (ref)					
	Yes	0.467	0.0003	0.089	1.595	1.337-1.896
Number of living child	1-3 (ref)					
	4-6	0.126	0.0004	0.101	1.134	0.934-1.388
	Above 6	0.217	0.0006	0.152	1.242	0.930-1.674
Source of drinking water	Unprotected (ref)					
	Protected	−0.206	0.0004	0.098	0.814	0.670-0.984
Random effect						
${\sigma}_{u0}^2$		1.295	0.0012	0.203		2.523-5.607
Random slope						
${\sigma}_{u11}^2$		0.725	0.0049	0.351		1.258-4.983
${\sigma}_{u21}^2$		0.502	0.0032	0.223		1.207-2.804

Note: *MC error* Monte Carlo error, *SD* Standard Deviation, *OR* Odds Ratio, *CI* Confidence Interval, $\kern0.50em {\sigma}_{u0}^2$ =regions variance, $\kern0.75em {\sigma}_{u11}^2$ =wealth index variance, ${\sigma}_{u21}^2$ =maternal working variance

This study also discovered that the source of drinking water has a significant impact on ARI. When compared to children who used an unprotected source of drinking water, children who used a protected source of drinking water were 18.6% less likely to have ARI.

Table 2 shows estimates of the variance of the random effect at the regional level as well, var. (*u*_0*j*_). As a result, Var (*u*_0*j*_) = 1.295 indicates that there was significant variation (which means the 95% credible intervals is greater than zero). This validated the significance of the regional difference in ARI incidence in Ethiopia’s regional state.

In this model, the researcher tested the variable that has a significant impact on the occurrence of ARI among children under the age of five in the intercept model by observing their respective region effect. As a result, regional level variables that are supposed to vary regionally, such as household wealth index and maternal working status, have been investigated.

The researcher discovered that the variance of the wealth index of the richer category slopes (${\sigma}_{u11}^2$ =0.725) with a credible interval of (95% CI: 0.223, 1.606) and the variance of the mothers currently working slopes (${\sigma}_{u21}^2$ = 0.502) with a credible interval of (95% CI: 0.188, 1.031) the interval was greater than zero. This suggests that the random slope of the household wealth index and the maternal working status for the incidence of ARI among children under the age of five varies by region.

## Discussion

The goal of this study was to find out what factors contribute to the prevalence of ARI in children under the age of five. According to the findings, the prevalence of ARIs in Ethiopian children varies greatly across regional states. The highest prevalence of ARI was found in Tigray and Oromia, at 15.3 and 14.4%, respectively, and the lowest in Benishangul Gumuz, at 2.6%, according to the descriptive data analysis. This result is consistent with [33–35]. Regional differences in sickness distributions could be due to a variety of factors; high-risk areas for ARI were observed in the country’s northern and central regions, particularly in Tigray and Oromia. Because these were highland areas, it was expected that there would be a high prevalence of ARI in children under the age of five. Another factor could be that many of the children in these areas were under the age of one-year-old, and the majority of these households relied on charcoal and cow dung for heat.

Based on their DIC, the Bayesian random coefficient model performed better in fitting the data appropriately. The analysis revealed that children aged 48–59 months have a lower risk of ARI than children aged less than 6 months, implying that as a child’s age increases, so does the risk of ARI. This result is in line with research conducted in Ahmadabad City and other low- and middle-income countries [22, 36] confirmed this result. This is due to the fact that as a child grows older, their immunity grows stronger and they are better able to resist infections such as respiratory infections.

The educational status of the mother was strongly related to ARIs. The findings show that more educated mothers are less fatalistic about their children’s illnesses, are more capable of seeking available health facilities, and have a significant impact on the traditional balance of family relationships, which has a significant impact on childcare. This means that caregivers with a high level of education were more likely to seek appropriate care for their child, which is consistent with previous research [33, 37–39].

In Ethiopia, a mother’s working status is an important determinant of the incidence of ARI among children under the age of five. Children with a working mother have lower ARI than those who do not work. The findings of our study agree with those of Jabessa and others [33, 40].

The household wealth index is another important factor in the occurrence of ARI. When compared to a lower wealth index, a higher wealth index reduces the likelihood of ARI occurrence. This finding is also consistent with studies conducted in Uganda, which found that the likelihood of ARI occurrence was reduced by 5 -18%, as confirmed by [41]. It is well known that families with a higher socioeconomic status are more likely to drink piped water and use sanitary toilets in low-income countries.

A child who had diarrhea was 28.4% more likely to have low ARI than a child who did not have diarrhea. This demonstrates that diarrhea has a negative impact on ARI in children under the age of five [42]. Children who were chronically malnourished were 59.5% more likely than children under the age of five who were not malnourished to have high ARI. The possible reason could be that malnutrition impairs immune system function and can increase the severity, duration, and susceptibility to acute respiratory infection, or that a child may have been exposed to higher ARIs due to a lack of adequate food, improper treatment, and insufficient care [22, 41].

A child who had recently received vitamin A was 16.6% less likely to suffer from ARI than a child who had not recently received vitamin A. Promoting Vitamin A supplementation for all children may improve children’s health. Several studies [43, 44] corroborated our findings. One of the other important determinants of the occurrence of ARIs was drinking water. Children who use a protected source of drinking water are 18.6% less likely to die than those who use an unprotected source of drinking water. According to the literature on source of drinking water, the main causes of diarrhea are source of drinking water [45, 46].

Another goal of the study was to identify regional variation, and our findings indicate that the Bayesian multilevel coefficient model revealed regional differences in ARI prevalence. According to the model adequacy results, the best-fitting model is the Bayesian multilevel logistic random coefficient model [47].

This research has both strengths and weaknesses. Using nationally representative samples, this study investigated the prevalence of ARIs among Ethiopian children under the age of five, as well as its relationship with demographic, health, environmental, and household socioeconomic characteristics. The findings of this study should help policymakers better understand this rapidly developing health problem and take appropriate policy actions. Aside from our significant contributions, our research has a number of flaws. For starters, rather than being measured clinically, the symptoms of ARIs were reported by mothers. The variables are subject to reporting bias because they were self-reported. Second, there was no data on household hygiene practices, which is an important predictor of infectious diseases in populations of all ages, and the data used for the study came from a cross-sectional survey, making it difficult to account for seasonal variations in child morbidity. Because there was no information on whether the children were sick, it’s possible that the fever and dyspnea symptoms were caused by something other than ARIs. The addition of sanitation variables, on the other hand, is expected to significantly reduce this disparity. Finally, because the data was secondary, no causal conclusions could be drawn about the correlations.

## Conclusions

The demographic, socioeconomic, health, nutritional, and environmental variables studied in this study were found to have a significant influence on ARI morbidity in children under the age of five. We discovered that children born in lower socioeconomic groups were more likely to develop ARI than children born in higher socioeconomic groups, and that a child who had diarrhea was more likely to develop ARI. It was also discovered that the prevalence of ARI among children under the age of five is lower in children whose mothers have a secondary or higher education level than in children whose mothers are uneducated, and that the risk of ARI decreases with the child’s age. The use of vitamin A supplements is another factor linked to an increase in the incidence of ARI in children under the age of five. Children who received vitamin A were less likely to develop ARI than children who did not receive vitamin A. Children who drank from a better source of water were less likely to get ARI than those who didn’t. Children born to working mothers had a lower ARI than children born to unemployed mothers. ARI was also more common in stunted children than in non-stunted children. The effect of regional variations in household wealth index and maternal working status implies that there are significant differences in ARI prevalence across regions, and a model with a random coefficient is more appropriate to explain the regional variation than a model with an empty and intercept model. There has been a considerable difference among regions through children ARI prevalence rate in Ethiopia.

ARI was prevalent in northern and central Ethiopia, particularly in Tigray and Oromia. These were high-altitude areas where ARI was common in children under the age of five. Children spend more time indoors when the weather is cold, exposing them to a wide range of bacteria, fungi, and viruses. When the temperature drops, flu, viruses, and bacteria become more stable in the air, forming respiratory droplets and damaging the airways, allowing bacteria to cause infections, most commonly ARI in the lungs. Another reason could be that there were a large number of children under the age of 1 year, and most households in these areas relied on charcoal and cow dung for fuel.

## Recommendations

The following recommendations can be made based on the findings and outcomes of this study: The level of education of mothers is critical in preventing ARIs in children. However, this requires a long-term investment. In the short term, health programs should prioritize assisting women who have little or no education. The government should create programs to improve the socioeconomic conditions of the poor; there is an obvious need for intervention to reduce economic inequalities and, ultimately, poverty among the populace. Higher-income households can afford better water and cooking fuel. To reduce ARI in children, the government must implement a comprehensive package of protective measures against diarrhea and a lack of Vitamin A supply, as well as other child care programs. Despite the fact that regional variation has been addressed, the distribution of ARI prevalence and the problem of identifying the hotspot-area are not addressed in this study. As a result, the researchers are encouraged to broaden their investigation by using spatial models.

## Supplementary Information

**Additional file 1.**

**Additional file 2.**

## Data Availability

The datasets generated and analyzed during the current study are available upon reasonable request from the corresponding author.

## Abbreviations

ARI: Acute Respiratory Infection
AIC/BIC: Akaike/Bayesian Information Criteria
CSA: Central Statistics Agency of Ethiopia
DIC: Deviance Information Criterion
EA: Enumeration Areas
EDHS: Ethiopian Demographic Health Survey
ICC: Intra class Correlation Coefficient
MCMC: Markov Chain Monte Carlo
OR: Odds Ratio
WHO: World Health Organization
