# Supplementary material for: Bayesian multilevel analysis of determinants of acute respiratory infection in children under the age of five years in Ethiopia
Source: BMC Pediatr. 2022 Mar 10;22:123. doi: 10.1186/s12887-022-03187-4 (PMC8908561; doi:10.1186/s12887-022-03187-4)
Supplement: Supplementary file 1 — Additional file 1. [file 12887_2022_3187_MOESM1_ESM.docx]

**Further Notes on Multilevel Models**

**Multilevel Logistic Regression Model**

The multilevel logistic regression extends from single level logistic regression model by including random effects to the model [1]. Suppose we have data consisting of children under five, (level one) grouped into regions (level two). Let $Y_{ij}$ be the binary response for ARI among $i^{th}$ under five years old children in region j and $X_{ij}$ be an explanatory variable. We define the probability of the response equal to one $\pi_{ij}=P(y_{ij}=1)$ Where; $\pi_{ij}$ be modeled using a logit link function. The standard assumption is that $Y_{ij}$ has a Bernoulli distribution. Then, the two-level models are given by:

$logit\left( \pi_{ij} \right)=\log\left[ \frac{\pi_{ij}}{1-\pi_{ij}} \right]$=$\beta_{0j}+\sum_{h=1}^{k} \beta_{hj}X_{hij}$………………………..………………….. (1)

$i=1,2\ldots\ldots n_{j}$,$h=1,2\ldots\ldots\ldots..k$,$j=1,2,\ldots\ldots\ldots11$

$\beta_{oj}=\beta_{o}+U_{oj}$,$\beta_{1j}=\beta_{1}+U_{1j},\ldots,\beta_{kj}=\beta_{k}+U_{kj}$

$logit\left( \pi_{ij} \right)=\log\left[ \frac{\pi_{ij}}{1-\pi_{ij}} \right]$=$\beta_{0}+\sum_{h=1}^{k} \beta_{hj}X_{hij}+U_{oj}+\sum_{h=1}^{k} U_{hj}X_{hij}$………………..……. (2)

$X_{i}=(X_{1ij,}X_{2ij,}\ldots\ldots\ldots.X_{kij})$ represent the first and the second level covariates, for variable k($\beta=\beta_{o},\beta_{1},\ldots\ldots\beta_{k})$ are the regression parameter coefficient. The parameters $U_{0j},U_{1j},\ldots\ldots..,U_{kj}$ is the random effect of the model parameter at level two. With the assumption $U_{hj}$, follows a normal distribution with mean zero and variance $\sigma_{u}^{2}$.

**Multilevel Analysis of Null Model**

The empty two-level model for a binary outcome variable refers to a population of groups (level-two units, i.e. regions)) and specifies the probability distribution for group-dependent probabilities without considering further explanatory variables [1,2]. This model only contains random groups and random variation within groups. It can be expressed with logit link function as follows.

$$logit\left( \pi_{ij} \right)=\beta_{0}+U_{0j} \ldots\ldots\ldots\ldots\ldots\ldots\ldots\ldots\ldots\ldots\ldots\ldots\ldots\ldots\ldots\ldots\ldots\ldots\ldots\ldots\ldots\ldots\ldots\ldots\ldots\ldots\ldots\ldots. (3)$$

$U_{0j}\sim IID(0,\sigma_{0}^{2}$)

Where $\beta_{0}$ indicates the population average of the transformed probability and $U_{0j}$ is the random deviations from this average for region j

The model decomposes the total variance into two-that of region and under five children, representing the between and within region variability’s in the ARI of under five children. The interclass correlation (ICC) measures correlation between observations within cluster as:

$$ICC=\frac{\sigma_{u}^{2}}{\sigma_{u}^{2}+\sigma_{e}^{2}} \ldots\ldots\ldots\ldots\ldots\ldots\ldots\ldots\ldots\ldots\ldots\ldots\ldots\ldots\ldots\ldots\ldots\ldots\ldots..\ldots\ldots\ldots\ldots\ldots\ldots\ldots\ldots\ldots\ldots\ldots\ldots(4)$$

**Multilevel Analysis of Random Intercept Model**

In the random intercept model the intercept is the only random effect meaning that the groups differ with respect to the average value of the response variable, but the relation between explanatory and response variables cannot be differ between groups [1]. The random intercept model expresses the log odds, $i.e$ the logit of $\pi_{ij}$ , as a sum of linear functions of the explanatory variables. That is,

$$logit\left( \pi_{ij} \right)=log\left[ \frac{\pi_{ij}}{1-\pi_{ij}} \right]=\beta_{0j}+\sum_{h=1}^{k} \beta_{h}X_{hij}\ldots\ldots\ldots\ldots.\ldots\ldots..\ldots\ldots\ldots..\ldots\ldots\ldots\ldots\ldots.. (5)$$

$$i=1,2,\ldots\ldots,n, j=1,2,\ldots\ldots11$$

Where the intercept term $\beta_{0j}$ is assumed to vary randomly and is given by the sum of an average intercept $\beta_{0}$ and group-dependent deviations;$\beta_{0j}=\beta_{0}+U_{0j}$.

As a result we have:

$$logit\left( \pi_{ij} \right)=\beta_{0}+\sum_{h=1}^{k} \beta_{h}X_{hij}+U_{0j}\ldots\ldots\ldots\ldots\ldots\ldots.\ldots\ldots\ldots\ldots\ldots\ldots\ldots..\ldots\ldots\ldots\ldots\ldots\ldots.. (6)$$

**Multilevel Analysis of Random Coefficients Model**

In the random coefficient model, both the intercepts and slopes are allowed to differ across the regions. Consider a model with group-specific regression of logit of the success probability logit ( $\pi_{ij}$) on a single level -one explanatory variable $X$

$$logit\left( \pi_{ij} \right)=\log\left[ \frac{\pi_{ij}}{1-\pi_{ij}} \right]=\beta_{0}+\sum_{h=1}^{k} \beta_{h}X_{hij}+U_{0j}+\sum_{h=1}^{k} U_{hj}x_{hij}\ldots\ldots\ldots\ldots\ldots\ldots(7)$$

The term $\sum_{h=1}^{k} U_{hj}x_{hij}$can be regarded as a random interaction between group and the explanatory variables. This model implies that two random effects characterize the groups: their intercepts and their slopes. It assumes that for different groups, the pairs of random effects ($U_{0}, U_{hj},h=1,2,\ldots,k)$ are independent and identical distributed. The random intercept variance,$Var\left( U_{0j} \right)=\sigma_{0}^{2}$, the random slope variance,$Var\left( U_{1j} \right)=\sigma_{1}^{2}$ and the covariance between the random effects $Cov\left( U_{0j};U_{1j} \right)=\sigma_{01}$ are called variance components [1].

**Bayesian Method of Parameter Estimation**

The models used in this paper are fitted using Markov Chain Monte Carlo (MCMC) based algorithms as implemented in the MLwiN [3]. Starting values for the fixed parameters was estimated from simpler models using a maximum likelihood approach, penalized quasi- likelihood (PQL) in MLwiN [4]. The simulated posterior distribution is then used to compute a point estimate and a confidence interval. The posterior distribution of *θ* is defined from Bayes’ theorem as:

$P(\theta|data) | P(data|\theta) P(\theta)$………………………………… ………… (8)

Here $P(\theta)$is the prior distribution for the parameter vector $\theta$ and should represent all
knowledge we have about $\theta$ prior to obtaining the data. In this study, we wish to express our prior ignorance in to the Bayesian system. This leads to non-informative priors. A non-informative prior distribution that is used to express complete ignorance of the value before the data is collected.

The default prior distribution applied in MLwiN when MCMC is used are ‘flat’ or ‘diffuse’ priors for all parameters [2]. The prior distributions for fixed effect parameter was $P\left( \beta\right)$~uniform distributions (1) and for random effect terms was,$P\left( \frac{1}{\sigma^{2}} \right)\sim Gamma(\alpha,\theta$) where $\alpha and \theta$ are fixed constant parameters.Let us denote the parameters $\beta_{0},\beta_{1},\ldots\ldots..,\beta_{k} andΩ_{u}$ as prior distributions would be given as follows;$P\left( \beta_{0} \right)\propto1,P\left( \beta_{1} \right)\propto1,\ldots\ldots,P\left( \beta_{k} \right)\propto1$and $P(Ω_{u})\propto$ inverse-Wishart$(m*S_{u},m)$distribution. The parameter $Ω_{u}$ is the variance-covariance matrices and $S_{u}$ is an estimate for the true value of $Ω_{u}$and $m$ is the number of row in the variance-covariance matrix.

The joint distribution of $n$ independent Bernoulli trials is the product of each Bernoulli densities, where the sum of independent and identically distributed Bernoulli trials has a Binomial distribution. Thus, the likelihood function is illustrated below as product of $n$ Bernoulli trials:

$L\left( \frac{\pi_{ij}}{y_{ij}} \right)=\prod_{ij} \left( \pi_{ij} \right)^{yij}(1-{\pi_{ij})}^{{1-y}_{ij}}$ and the logit functions are defined in the equation (7).

The $\pi_{ij}$ represents the probability of the event for child $i$ in region $j$ who has covariate vector $X_{ij}$ , $Y_{ij}=1$ indicates the presence (child having ARI) and $Y_{ij}=0$ the absence (child not having ARI) of ARI for the given subject.

The posterior distribution is obtained by multiplying the prior distribution over all
parameters by the full likelihood functions. The posterior distribution is:

$P\left( \beta_{h} | Ω_{u},U_{oj},y_{ij} \right)\propto\prod_{ij} {\pi_{ij}}^{y_{ij}}\left( 1-\pi_{ij} \right)^{1-y_{ij}}P(\beta)$…………………….………… (9)

Where $h=1,2,\ldots\ldots\ldots\ldots\ldots.k$ andthe full conditional distribution of the variance-covariance parameter $Ω_{u}$ has been given as:

$P(Ω_{u}$|$\beta_{h},U_{oj},Y_{ij}) \propto P(Y_{ij}|\beta_{h}, Ω_{u}, U_{oj})P(U_{oj}|Ω_{u})P\left( Ω_{u} \right)$……………………..……(10)

**Metropolis–Hastings algorithm**

The Metropolis–Hastings algorithm is a Markov chain Monte Carlo (MCMC) technique for obtaining a series of random samples from a probability distribution. A sequence of sample values is generated using the Metropolis–Hastings algorithm. The posterior does not resemble any distribution we are familiar with (no Conjugate), and some (or all) of the full conditionals do not resemble any distribution we are familiar with for individuals whose entire conditionals we are unfamiliar with (no Gibbs sampling). Because of this, we became interested in the Metropolis–Hastings algorithm.

**Model selection and comparison**

Model selection is the process of selecting the best model from a set of options based on the performance of the models. The DIC is a popular statistic for comparing models in a Bayesian context. Define the deviance as $D(\theta)$=$-2\log\left( p\left( y | \theta\right) \right)+c$, where *y* are the data, θ are the unknown parameters of the model and $p\left( y | \theta\right)$ is the likelihood function. C is a constant that cancels out in all calculations comparing different models and thus is not required to be known. The expectations $\hat{D}$ =$E[D\left( \theta\right)]$ is a measure of how well the model fits the data; the greater the value, the worse the fit. The larger this value, the better the model's fit to the data. The deviance information criterion is defined as DIC = $\hat{D}$+$pD$. Because D decreases as the number of parameters in a model increase, the pD term compensates for this effect by favoring models with fewer parameters. The advantage of DIC over other criteria for Bayesian model selection is that it can be easily calculated from samples generated by an MCMC simulation. AIC and BIC require calculating the likelihood at its maximum over, which is not readily available from the MCMC simulation. To calculate DIC, simply compute $\hat{D}$ as the average of $D(\theta)$ over a sample values of $\theta$, and $D(\hat{\theta})$ as the value of $D$ evaluated at the average of the samples of $\theta$ [5]. The DIC is then derived directly from these approximations.

**References**

[1] T.A.B. Snijders, R.J. Bosker, Multilevel analysis Sage Publications, Thousand Oaks, CA. (1999).

[2] H. Goldstein, Multilevel statistical models, John Wiley & Sons, 2011.

[3] J. Rasbash, W. Browne, M. Healy, B. Cameron, C. Charlton, MLwiN version 2.02 [Computer software], Bristol, Engl. Cent. Multilevel Model. (2005).

[4] J. Rasbash, C. Charlton, K. Jones, R. Pillinger, Manual supplement for MLwiN version 2.26, Bristol Cent. Multilevel Model. Univ. Bristol. (2012).

[5] V. Bewick, L. Cheek, J. Ball, Statistics review 14: Logistic regression, Crit. Care. 9 (2005) 1–7.
