# Supplementary material for: Bayesian multilevel analysis of determinants of acute respiratory infection in children under the age of five years in Ethiopia
Source: BMC Pediatr. 2022 Mar 10;22:123. doi: 10.1186/s12887-022-03187-4 (PMC8908561; doi:10.1186/s12887-022-03187-4)
Supplement: Supplementary file 2 — Additional file 2. [file 12887_2022_3187_MOESM2_ESM.docx]

**Results of Bayesian Multilevel Analysis**

**Model comparison of Bayesian Multilevel logistic regressions**

From Table A1 we see that the comparison of the fit of Bayesian multilevel logistic regression models using the summary of the fitted model. The model, which has small $DIC$, is the best model for the data set, so the result, shows that model 3 was an improved fit as compared to the rest models in any combination of variables in the data set. The average deviance from the complete set of iterations ($\hat{D}$) also decreased from model 1 to model 2 and from model 2 to model 3. $D(\hat{\theta})$ shows that the deviance at the expected value of the unknown parameters and it also shows the decreasing trend from model 1 to model 2 and from model 2 to model 3. Also the model complexity is measured by $pD$ (The effective number of parameters in the model), the larger the $pD$ is easier to fit the data. Based on this fact, the third model has the largest value of this measure, it is selected again.

**Table A1**: DIC values for model comparisons

| **Model** | $\hat{\boldsymbol{D}}$ | $\boldsymbol{D}\boldsymbol{(}\hat{\boldsymbol{\theta}}\boldsymbol{)}$ | $\boldsymbol{Pd}$ | $\boldsymbol{DIC}$ |
| --- | --- | --- | --- | --- |
| Model 1 | 5097.64 | 4848.33 | 249.31 | 5346.95 |
| Model 2 | 4699.42 | 4427.04 | 272.38 | 4971.79 |
| Model 3 | 4612.55 | 4275.15 | 337.40 | 4949.95 |

Note: Model 1: null model; model 2: random intercept model; model 3: random coefficient model, **DIC:** Deviance Information Criterion , $\hat{\boldsymbol{D}}$ **:** is the posterior mean of the deviance, measuring how well a model fits the data, $\boldsymbol{D(}\hat{\boldsymbol{\theta}}\boldsymbol{)}$: The deviance at the expected value of the unknown parameters, $\boldsymbol{pD}$ **:** is the effective number of parameters measuring model complexity.

**Result of Bayesian Multilevel Empty Model**

From Table A2, the between-region (level two) variance of ARI status of under five children was found to be significant because the credible interval of the respective parameters was greater than zero.

In order to get an idea of how much of the variation in ARI status of among children under five was attributable to the region level factors, it is useful to see the intra-region correlation coefficients $(ICC)$, so the $ICC$ was $0.233$, which measures the proportion of variance of the ARI of children under five that is between regions, not within regions. This means that around 23.3% of the variance in ARI of under five children are due to variation between regions. Whereas the remaining 76.7% attributable to individual level, i.e., within regional differences.

**Table A2:** Posterior summaries for parameters of the empty model

| Model | Posterior mean | MC error | SD | 2.5% | 50% | 97.5% |
| --- | --- | --- | --- | --- | --- | --- |
| Fixed intercept($\beta_{0}$) | -2.789 | 0.0003 | 0.072 | -2.936 | -2.789 | -2.652 |
| Random intercept var($U_{0j}$) | 0.998 | 0.0006 | 0.136 | 0.750 | 0.989 | 1.279 |

### Note: MC error: Monte Carlo error, SD: Standard deviations

**Result of Random Intercept Bayesian Multilevel Model**

The result of intercept model displayed in (Table A3) also estimates that, the variance of random effect at the regional level var ($u_{0j}$) =1.107 since the 95% credible interval was greater than zero under the interval, which indicates that there is a significant regional variation.

**Table A3:** Bayesian Multilevel Logistic Regression of Random Intercept

| Fixed effect | | | | | | | |
| --- | --- | --- | --- | --- | --- | --- | --- |
| Variables | Category | Posterior mean | SD | MC error | 2.5% | 50% | 97.5% |
| - | Intercept | -3.280 | 0.367 | 0.0063 | -4.035 | -3.270 | -2.563 |
| Sex | Male(ref) |  |  |  |  |  |  |
|  | Female | 0.007 | 0.080 | 0.0003 | -0.151 | 0.008 | 0.170 |
| Child age | <6 (ref) |  |  |  |  |  |  |
|  | 6-11 | 0.245 | 0.162 | 0.0007 | -0.066 | 0.243 | 0.563 |
|  | 12-23 | 0.087 | 0.147 | 0.0007 | -0.195 | 0.088 | 0.369 |
|  | 24-35 | -0.177 | 0.169 | 0.0009 | -0.509 | -0.180 | 0.153 |
|  | 36-47 | -0.193 | 0.186 | 0.001 | -0.556 | -0.193 | 0.176 |
|  | 48-59 | -0.539 | 0.196 | 0.001 | -0.925 | -0.539 | -0.148 |
| Maternal age | 15-19(ref) |  |  |  |  |  |  |
|  | 20-34 | 0.153 | 0.223 | 0.0022 | -0.264 | 0.146 | 0.626 |
|  | 35-49 | 0.050 | 0.249 | 0.0021 | -0.419 | 0.043 | 0.556 |
| Wealth index of household | Poorest (ref) |  |  |  |  |  |  |
|  | Poorer | -0.214 | 0.124 | 0.0005 | -0.459 | -0.214 | 0.024 |
|  | Middle | -0.609 | 0.145 | 0.0005 | -0.892 | -0.609 | -0.331 |
|  | Richer | -0.812 | 0.161 | 0.0006 | -1.130 | -0.813 | -0.509 |
|  | Richest | -0.598 | 0.204 | 0.0009 | -0.994 | -0.595 | -0.213 |
| Place of residence | Rural (ref) |  |  |  |  |  |  |
|  | Urban | 0.244 | 0.220 | 0.001 | -0.194 | 0.245 | 0.684 |
| Breast feeding | Never breast feed(ref) |  |  |  |  |  |  |
|  | Ever breast feed, not currently | 0.229 | 0.253 | 0.0025 | -0.249 | 0.219 | 0.737 |
|  | Still breast feed | 0.356 | 0.258 | 0.0028 | -0.150 | 0.349 | 0.868 |
| Maternal  Education | No education(ref) |  |  |  |  |  |  |
|  | Primary | 0.154 | 0.105 | 0.0004 | -0.051 | 0.154 | 0.362 |
|  | Secondary and higher | -0.449 | 0.201 | 0.0007 | -0.851 | -0.443 | -0.065 |
| Vitamin A Supplement | No (ref) |  |  |  |  |  |  |
|  | Yes | -0.183 | 0.088 | 0.0003 | -0.355 | -0.184 | -0.010 |
| Had diarrhea Recently | No(ref) |  |  |  |  |  |  |
|  | Yes | 1.435 | 0.096 | 0.0003 | 1.245 | 1.590 | 1.621 |
| Fuel type | Unclean/unsafe(ref) |  |  |  |  |  |  |
|  | Safe/clean | -0.065 | 0.266 | 0.0009 | -0.587 | -0.061 | 0.454 |
| Maternal Work | Not working(ref) |  |  |  |  |  |  |
|  | Working | 0.268 | 0.094 | 0.0003 | 0.084 | 0.268 | 0.459 |
| Wasting | No(ref) |  |  |  |  |  |  |
|  | Yes | 0.122 | 0.120 | 0.0004 | -0.116 | 0.119 | 0.354 |
| Stunting | No(ref) |  |  |  |  |  |  |
|  | Yes | 0.456 | 0.087 | 0.0003 | 0.292 | 0.456 | 0.632 |
| Number of living child | 1-3 (ref) |  |  |  |  |  |  |
|  | 4-6 | 0.126 | 0.100 | 0.0004 | -0.070 | 0.123 | 0.319 |
|  | Above 6 | 0.212 | 0.150 | 0.0006 | -0.088 | 0.213 | 0.499 |
| Source of drinking water | Unprotected(ref) |  |  |  |  |  |  |
|  | Protected | -0.203 | 0.095 | 0.0003 | -0.387 | -0.202 | -0.016 |
| Random effect | | | | | | | |
| Var ($u_{0j}$) = $\sigma_{u0}^{2}$ | | 1.107 | 0.158 | 0.0007 | 0.829 | 1.095 | 1.456 |

**Note**: MC error: Monte Carlo error, SD: Standard Deviation,$\sigma_{u0}^{2}$**=**regions variance

**The plots of Convergence test of wealth index of household and diarrhea status of under five children.**


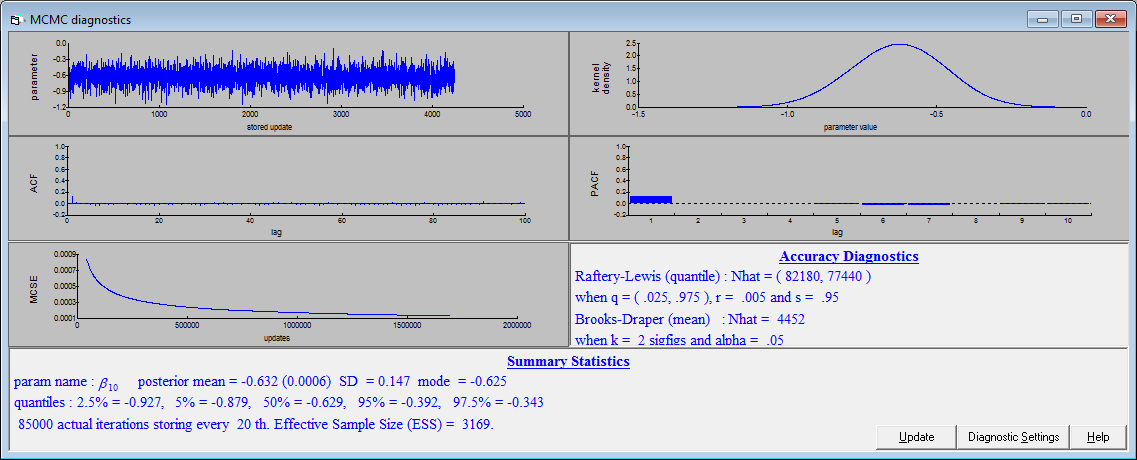


**Figure 1:** Plots of wealth index of household in middle class


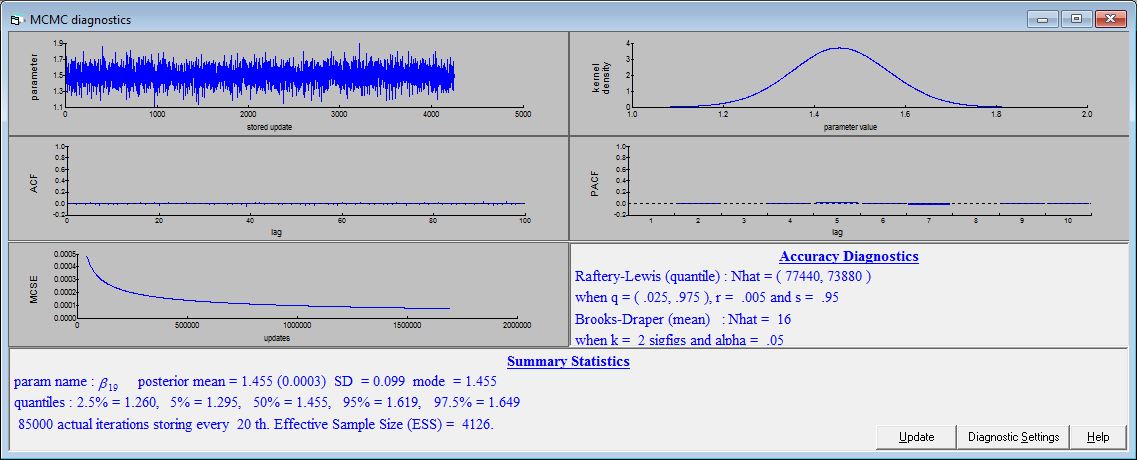


**Figure 2**: Plots of a child had diarrhea recently
